# Preassembled complexes of hAgo2 and ssRNA delivered by nanoparticles: a novel silencing gene expression approach overcoming the absence of the canonical pathway of siRNA processing in the apicomplexan parasite Babesia microti, blood parasite of veterinary and zoonotic importance

**DOI:** 10.1080/22221751.2024.2438658

**Published:** 2024-12-09

**Authors:** Shimaa A. E-S. El-Sayed, Mohamed A. Rizk, Hang Li, Uday Kumar Mohanta, Iqra Zafar, Shengwei Ji, Zhuowei Ma, Thom Do, Yongchang Li, Daisuke Kondoh, Jerzy Jaroszewski, Xuenan Xuan

**Affiliations:** a National Research Center for Protozoan Diseases, Obihiro University of Agriculture and Veterinary Medicine, Obihiro, Japan; b Department of Biochemistry and Molecular Biology, Faculty of Veterinary Medicine, Mansoura University, Mansoura, Egypt; c Department of Internal Medicine and Infectious Diseases, Faculty of Veterinary Medicine, Mansoura University, Mansoura, Egypt; d Livestock and Dairy Development Department, Veterinary Research Institute, Lahore, Pakistan; e Department of Veterinary Medicine, Agriculture College of Yanbian University, Yanji, People’s Republic of China; f Department of Veterinary Medicine, Obihiro University of Agriculture and Veterinary Medicine, Obihiro, Japan; g Department of Pharmacology and Toxicology, Faculty of Veterinary Medicine, University of Warmia and Mazury in Olsztyn, Olsztyn, Poland; h Research Center for Asian Infectious Diseases, The Institute of Medical Science, The University of Tokyo, Minato-ku, Japan

**Keywords:** *Babesia*, gene delivery system, chitosan nanoparticles, RNAi, noncanonical pathway

## Abstract

Due to the lack of efficacy of the currently used chemical drugs, poor tick control, and lack of effective vaccines against *Babesia*, novel control strategies are urgently needed. In this regard, searching for anti-*Babesia* gene therapy may facilitate the control of this infection. Following this pattern, small interfering RNAs (siRNAs) are widely used to study gene function and hence open the way to control the parasite. However, the primary constraint of this approach is the lack of *Babesia* to RNA-induced silencing complex (RISC) enzymes, making siRNA impractical. In this study, we preassembled complexes with the human enzyme argonaute 2 (hAgo2) and a small interfering RNA (siRNA)*/*single-stranded RNA (ssRNA) against *B. gibsoni* and *B. microti* metabolite transporters. The assembled complexes were generated by developing a gene delivery system with chitosan dehydroascorbic acid nanoparticles. The delivery system effectively protected the loaded RNAi and targeted *Babesia-*infected RBCs with a relatively high internalization rate. The assembled complexes were successfully transfected into live parasites for specific slicing of *Babesia* targets. We demonstrated a reduction in the expression of target genes at the mRNA level. Furthermore, this silencing inhibited *Babesia* growth *in vitro* and *in vivo*. For the first time, we used this method to confirm the role of the assembled complexes in manipulating the noncanonical pathway of RNAi in *Babesia* parasites. This novel method provides a means of silencing *Babesia* genes to study their role in host–parasite interactions and as potential targets for gene therapy and control.

## Introduction

Babesiosis is a disease that spreads worldwide among both human and animals and has high economic and welfare impacts. *Babesia microti* (*B. microti*) is the main etiologic agent recognized for babesiosis in humans in the USA [1]. The infection in dogs is mainly caused by *Babesia gibsoni* (*B. gibsoni*) or *B. canis* [2]. *B. gibsoni* has considerable endemic importance in various American states and Asia counties, like Japan [2]. This high endemic importance of the infection might be attributed to the absence of an effective control strategy. Taken together, the current therapies not only have toxic side effect, also have drug tolerance [2]. This issue spurred interest in developing a novel and more effective strategy to suppress the growth of *Babesia* into the host’s erythrocytes. To that end, the pathology of babesiosis depends on the development of parasitaemia, which improves as a sequela to the cyclical replication of *Babesia* parasites in the host's RBCs [3]. For rapid growth in infected erythrocytes, *Babesia* species need enough substrates to fuel their vigorous metabolism. Hence, the parasite prepares the host erythrocyte by inducing a specialized transporter for the uptake and removal of metabolites, which is significantly different from the host cell transporter [4]. Subsequently, targeting these overexpressed transporters on the surface of infected RBCs (iRBCs) is a critical step in inhibiting parasite growth; therefore, these transporters represent potential targets for novel therapeutic interventions. Indeed, very few studies are available on the transporters that are required for the uptake and removal of metabolites from *B. gibsoni* parasites into iRBCs. Therefore, molecular identification of these transporters and exploration of their functions will help significantly reduce the growth and survival of *Babesia* by interfering with the expression of these transporters via the use of silencing RNA (siRNA). To accomplish this silencing process, in the present study, for the first time, we developed a safe and effective gene delivery system targeting the noncanonical pathway for gene interference in *Babesia* species (Figure S1). In this regard, nanoparticles are recommended owing to their small size, ability to overcome biological barriers, and specific gene delivery potency [5]. Following this pattern, chitosan (Cs) nanoparticles have been suggested due to their history of safe use as construction materials in a large variety of parenteral delivery systems [6]. Various approaches, in a parasite biologically related to *Babesia, Plasmodium,* have been demonstrated to target glucose transporter (GLUT-1) using Cs nanoparticles bearing glucosamine as a ligand of the hexose transporter to discriminate between *Plasmodium falciparum* (*Pf*)*-*iRBCs and noninfected RBCs [6]. The results showed the advantages of this strategy in parasite killing *in vivo* [6]. Interestingly, a previous study [7] reported the superiority of dehydroascorbic acid (DHA), a ligand for GLUT-1, over other ligands used to discriminate between *Pf-*iRBCs and noninfected RBCs. Therefore, we can hypothesize that nanometer-sized particles bearing DHA may preferentially target iRBCs and deliver a large amount of the desired medication at the site of action. Subsequently, in the present study, we used CsDHA nanoparticles as a potential biomolecule delivery system that suppose to efficiently deliver therapeutic nucleic acid molecules to *B. gibsoni*-infected erythrocytes *in vitro* and to *B. microti*-infected erythrocytes *in vivo* (Figure S1). Therefore, the present study aimed to (i) identify specialized transporters such as nitrite, P-type ATPase, and V-type ATPase induced by *B. gibsoni* on the surface of the parasite membrane for the uptake and removal of parasite metabolites; (ii) establish a novel chitosan–dehydroascorbic acid–nanoparticle (CsDHA) gene delivery system for suppressing the growth of *B. gibsoni* with efficient protection of therapeutic nucleic acid molecules from rapid degradation, significantly targeting iRBCs with greater uptake and substantial cell target selectivity, with minimal cytotoxicity to mammalian cells; and (iii) investigate the inhibitory effect of CsDHA carrying RNAi (small interfering RNA (siRNA) and single stranded RNA (ssRNA) alone or preassembled to human Ago 2 protein to target nitrite, P-type ATPase, and V-type ATPase transporters noncanonically against the growth of *B. gibsoni* in *in vitro* culture and in an experimental animal model infected with *B. microti.*

## Materials and methods

### Chitosan production and characterization

Chitosan preparation is divided into three consecutive steps: demineralization of shrimp shells, chitin processing (deproteinization), and chitosan processing (deacetylation) [8]. The protein content of the samples was evaluated through the Bradford method [9]. The percentage of deproteinization (DP) was calculated according to the following equation: DP (%) = [(P1 − P2)/P1] × 100%, where P1 and P2 represent the protein content of the crude shrimp shells and prepared chitin, respectively. The degree of acetylation was calculated by potentiometric titration using the following equation: NH2% = 16.1×(y − x)/M (2), where M is the weight of the chitosan used, x is the first inflection point on the graph of the measured pH vs. the titration volume, and y is the second inflection point. The inflection points of the curve were determined according to [10]. The steps of each procedure are detailed in (supplementary materials 1).

### Cs-DHA-RNAi-loaded nanoparticles

Cs- and CsDHA-loaded nanoparticles were prepared via ionic gelation methods [11]. Cs was dissolved in 1% v/v glacial acetic acid to create Cs solutions (0.5% w/v). TPP (the crosslinking agent) was dissolved in deionized water (2% w/v). Then, 1.2 mL of TPP was added to 3 mL of the Cs solution at room temperature and continuously stirred at 700 rpm for 30 min by a magnetic stirrer to create the Cs nanoparticles. The nanoparticles were subsequently incubated for another 30 min at room temperature, followed by centrifugation (Himac CF 7D2 Ultracentrifuge, HITTACHI, Japan) at 10,000 rpm at 10°C for 30 min to collect the nanoparticles. The supernatants were discarded, and the pellets of nanoparticles were resuspended in filtered (0.25-μm Invitrogen, USA) deionized water. For the synthesis of CsDHA-loaded nanoparticles, a DHA solution was prepared by dissolving 200 mg of DHA in 4 ml of DMSO (0.05%). Three milliliters of DHA solution was mixed with 3 ml of Cs solution and stirred for 30 minutes before the addition of 1.2 mL of TPP solution.

To bind either siRNA or ssRNA to the CsDHA nanoparticles, we tested two different techniques: entrapment and adsorption. In the entrapment technique, 3 μL (5 μM/μL) of siRNAs (designed for the *B. gibsoni* nitrite format transporter NFT) (Thermo Fisher design tool) was added to 1.2 mL of TPP solution (2% w/v), and this mixture was added to the DHA/Cs. The produced solution was constantly magnetically stirred (700 rpm) at room temperature. For the adsorption technique, the CsDHA-TPP nanoparticles were synthesized first and then ten microliters (5 μM/μL) of NFT siRNA was mixed with 10 μL of the suspended nanoparticles. Then, the obtained product was quickly mixed and incubated for 2 hr at RT before further analysis [12].

### siRNA, ssRNA+hago2 complex loading, and nanoparticle morphological analysis

For ssRNA+hAgo2 and siRNA+hAgo2 complexes, we used hAgo2 (Sino-Biological, Beijing, China) ssRNA and siRNA designed from *B. gibsoni* targets with defined functions (nitrite format transporter) and only ssRNA for p-type ATPase and v-type ATPase transporters. These transporters were identified from the whole *B. gibsoni* genome sequence (data not shown). The siRNA NFT was designed as previously described, while ssRNAs were designed by using an RNA design tool (Integrated DNA Technologies, Coralville, Iowa). The selected ssRNAs (Table S1) were synthesized with modifications, including 5′P-uN-(21–23)-dTT-3′. We assembled the complexes with modifications [13]. Briefly, for assembly, we used 250 ng-100 µg of hAgo2 and 5 µM NTF ssRNA (19-21 nucleotides) in 14 µL of assembly buffer (30 mM HEPES, pH 7.4, 150 mM KOAc, and 2 mM MgCl_2_). We incubated the sample for 90 minutes at room temperature. The formation of complexes was evaluated by an electrophoretic mobility shift assay (EMSA) using 4% agarose gels [14]. A total of 100 µg of hAgo2 and 5 µM of ssRNA/siRNA had a significant effect on the assembly and strong binding between hAgo2 and the RNAi complexes because this ratio was chosen for the assembly of all the complexes and the loading of the complexes into the CsDHA NPs via the entrapment method, as previously described.

Transmission electron microscopy (TEM) was used to measure the mean particle size of the newly synthesized empty Cs and CsDHA-siRNA/ssRNA+hAgo2-loaded nanoparticles. The samples were collected by centrifugation and resuspended in deionized distilled water (0.25 μm, Invitrogen, USA). On the TEM copper microgrid, a drop of a dispersed nanoparticle was applied and kept for 5–10 minutes to evaporate at ambient temperature (25 ± 2°C) and then imaged via TEM [12].

### Entrapment efficiency

The entrapment efficiency of the Cs nanoparticles for DHA, siRNA, ssRNA, and hAgo2 was measured by quantifying the free DHA, siRNA, ssRNA, and hAgo2 in the supernatant recovered from centrifugation (10,000 rpm at 10°C for 30 min) after 1, 2, and 3 h and measuring the absorbance at wavelengths of 210, 260, and 280 nm, respectively detailed in (supplementary materials 1).

### Gel retardation assay and stability studies

Using 4% w/v agarose gel electrophoresis and ethidium bromide staining (Invitrogen, Carlsbad, CA, USA), the effectiveness of siRNA binding to the Cs-TPP nanoparticles was assessed. The wells were filled with 20 μL of sample (adsorbed and entrapment techniques) containing 0.2 μg of siRNA. The size reference utilized was a 10-bp DNA ladder. Then, siRNA-loaded CsDHA nanoparticles prepared via the entrapment method were selected for use in a serum protection assay because of their small particle size and high entrapment efficiency. Two hundred microliters of the Cs nanoparticles loaded with siRNA (5 μM siRNA) were incubated at 37°C in the corresponding volume of RPMI supplemented with 10% canine serum. As a control, naked siRNA was subjected to the same procedure. To perform gel electrophoresis, 40 μL of the mixture was removed and stored at -20°C for 30 min, 2 h, 4 h, 24 h, 4 days, 6 days, or 7 days. To stop serum activity, the samples were incubated for 3 minutes at 60°C in a bath incubator before gel electrophoresis. After that, 5 μL of heparin (1,000 U/mL) was injected to displace the siRNA from the CsDHA nanoparticles. The integrity of the siRNA displaced from the nanoparticles was analysed by conducting gel electrophoresis with a 4% w/v agarose gel stained with ethidium. Electrophoresis was performed for 30 min at 110 volts [12].

### *In vitro* release studies and determination of the hemolytic and cytotoxic effects of the developed NPs

The release characteristics of DHA and siRNA, ssRNA, and hAgo2 were studied using PBS at different pH values (pH 7.4, 9, and 6.4) to mimic the normal environment in the blood and inside the endosome, respectively. Moreover, the synthesized NPs were incubated with GSH (10 mM) to mimic the environment inside the targeted *B. gibsoni*-infected RBCs detailed in (supplementary materials 1).

Hemolysis assays were performed according to [15]. The cytotoxicity assay for the developed NPs was performed following the instructions of the Cell Counting Kit-8 (CCK-8, Japan) detailed in (supplementary materials 1).

### Cellular internalization

FITC-labelled –CsDHA-siRNA NPs were synthesized as follows: a FITC solution was prepared by dispersing 1 mg of FITC powder into 1 mL of DMSO. Approximately 50 µL of FITC was then added to 200 µL of DHA solution, and the mixture was incubated for 20 minutes in the dark at 24°C. The mixture of FITC–DHA was then added to 600 µL of Cs solution (0.5%) and further incubated for 15 minutes. A volume of 250 µL of TPP (2%) containing 3 μl of siRNA (5 μM) was added to 850 µL of the total mixture and was thoroughly mixed to ensure that a homogeneous suspension was obtained. The FITC-DHA-siRNA-encapsulated Cs NPs were then stored at 4°C for further analyses. After that, fluorescence microscopy and flow cytometry were used [16,17]. All procedures are detailed in (supplementary materials 1).

### Transfection of parasites

Babesia gibsoni iRBCs with 1% parasitemia and 10% HTC were used in this experiment. To show protein transfection of the iRBCs, we used an anti-His tagged antibody to detect hAgo2- and Cya5-labelled ssRNA. For transfections, the iRBCs were incubated with 10 μg/ml CsDHA NPs (5 μM ssRNA+100 μg hAgo2) for 4 hours at 37°C to increase permeability and improve transfection into iRBCs. After incubation, the samples were concentrated by centrifugation (at 1,500 rpm for 10 minutes) and washed with phosphate-buffered saline (PBS). Parasite lysates from transfected and nontransfected cultures were prepared and subjected to western blotting to detect protein transfection, while ssRNA transfection was determined via fluorescence microscopy, flow cytometry, and fluorescence intensity, as previously described.

### *In vitro* inhibition and quantitative polymerase chain reaction (qPCR) assays

The parasites were harvested when the parasitemia reached more than 5% and diluted with fresh canine RBCs to 1%. Then, 10 μg/mL CsDHA NPs were loaded with siRNA 1 (5 μM siRNA NFT), Cs NP complex 1 (5 μM siRNA NFT+100 μg hAgo2), ssRNAs 1, 2, and 3 (5 μM ssRNA) (ssRNA NFT, v-type ATPase, p-type ATPase, respectively), and Cs NP complexes 2, 3, and 4 (10 μg/ml CsDHA NPs (5 μM ssRNA+100 μg hAgo2) (ssRNA NFT+ hAgo2, v-type ATPase+ hAgo2, and p-type ATPase+ hAgo2, respectively). Additionally, naked siRNA NFs were incubated with iRBCs (1% parasitemia) or 10% HTC for 4 days, and the medium was replaced daily. After the fourth day, a Giemsa-stained slide was prepared for each complex and examined with a light microscope.

We evaluated silencing by measuring the reduction in messenger RNA (mRNA) by qPCR. RNA was isolated from the transfected parasites after 48hrs using TRIzol. The concentration of the RNA was evaluated by spectrophotometry (NANO DROP 2000). We used 100 ng of total RNA for cDNA synthesis (super script III first strand, Invitrogen, USA) (detailed in additional file 1). The primers used are listed in Table 1.

### Lactate assay

The silencing effect of hAgo2+ssRNA against the *B. gibsoni* nitrate format transporter was confirmed by measuring the lactate concentration (CheKine Micro Lactate Assay Kit, My BioSource, California, San Diego, USA) inside the RBC cytosol and parasite cytosol. The transfected *B. gibsoni*-infected RBCs were collected after 8 hours of transfection and washed with PBS 3 times with subsequent centrifugation at 1500 rpm for 15 minutes. The supernatant was collected to measure the lactate concentration in the culture medium, and the pelleted RBCs were lysed with 1% saponin for 3 minutes. The lysed mixture was centrifuged at 1500 rpm for 15 min, and the supernatant was collected to measure the lactate concentration in the RBC cytosol. The pelleted parasites were lysed with 1% saponin for 3 min to collect the parasite cytosol. The samples were centrifuged as previously described, and the supernatant was collected to measure the lactate concentration in the parasite cytosol. *B. gibsoni* non transfected RBCs were used as a control.

### *In vivo* biodistribution and inhibition

Five groups of BALB/c mice (n = 5 per group) aged 8 weeks (CLEA, Tokyo, Japan) were injected intraperitoneally with 1 × 10^7^ *B. microti* Peabody mjr (ATCC® PRA-99™) iRBCs [18], except for the mice in the first group, which remained uninfected and served as a negative control. When infected mice demonstrated 1% parasitemia, CsDHA NPs (10 μg/mL) loaded with ssRNA against *B. microti* NFT and assembled with hAgo2 (5 μm+100 μg/mL) were injected into mice in one experimental group I/V daily for 7 successive days. The second group was treated with CsDHA NPs at 10 μg/mL I/V daily for 7 successive days. The third group was not subjected to treatment and served as a positive control group. The fourth group was given the commonly used antibacterial control drug, DA, intraperitoneally at a dosage of 25 mg/kg. A Giemsa-stained blood smears were prepared from tail vein tail blood every 48 h until 30 days postinoculation. Following the completion of the study, all of the mice were euthanized humanely via inhalation of the chemical chloroform, which was followed by neck dislocation (physical euthanasia). For biodistribution, 2 BALB/c mice aged 8 weeks were injected intraperitoneally with 1 × 10^7^ *B. microti.* When infected mice demonstrated 25% parasitemia, FitC-labelled CsDHA NPs (10 μg/mL) loaded with ssRNA against *B. microti* NFT and assembled with hAgo2 (5 μm+100 μg/mL) were injected into the mice I/V. After 24 hr, blood was collected through heart puncture after the mice were euthanized via the inhalation of chemical chloroform. Then, neck dislocation (physical euthanasia) was applied, and different organs were collected (liver, kidney, lung, heart, and spleen). Blood and different organs were processed for flow cytometry [19] to determine the amount of internalized NPs in each organ.

### Statistical analysis

Using GraphPad Prism, one-way ANOVA was used to determine significant differences between the studied groups (version 5.0 for Windows; GraphPad Software, Inc., San Diego, CA, USA). A *P* value less than 0.05 was considered to indicate statistical significance.

## Results

### Cs NPs exhibit high entrapment efficiency

Chitosan was successfully extracted from shrimp cells (Figure S2A) with 77.74% and 75% DD and DP, respectively (Figure S2B). The extracted chitosan was then used in the synthesis of Cs NPs. The mean particle sizes of the CsDHA-TPP- and siRNA-loaded CsDHA-TPP nanoparticles ranged from 80-100 nm, as detected by TEM (Figure S2C). Moreover, a decrease in particle size was observed after the loading of siRNA into the CsDHA-TPP nanoparticles in comparison to that of the unloaded Cs-DHA-TPP nanoparticles. In addition, TEM images of the CsDHA-TPP nanoparticles unloaded and loaded with siRNA revealed an assorted morphology consisting mostly of spherical and irregular particles (Figure S2C).

Importantly, a higher entrapment efficiency of the Cs NPs for both the loaded DHA and the siRNA were observed with increasing incubation period and reached 86% and 94%, respectively, after 3 h of incubation (Figure S1D).

### Entrapped siRNA binds to cs NPs

**Figure 1.**
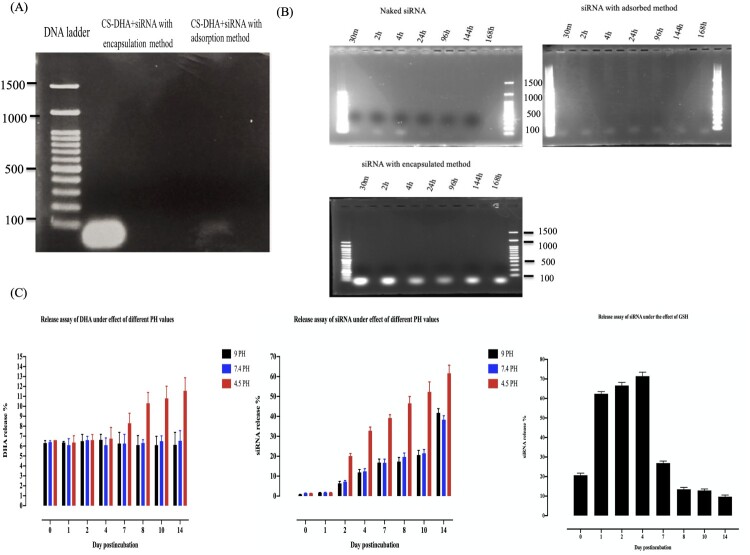
Stability studies of the synthesized Cs NPs. (A) Gel retardation assay with 4% agarose gel electrophoresis. (B) Serum protection assay of Cs NPs for the loaded siRNA with entrapment and adsorption methods. (C) *In vitro* release assay of the loaded DHA and siRNA under different pH conditions and GSH concentrations.

To further investigate siRNA binding to the CsDHA-TPP nanoparticles, we assessed two different methods of loading (entrapment and adsorption), and the binding efficiency of each method was confirmed via agarose gel electrophoresis. For CsDHA-TPP-siRNA loaded via the entrapment method, binding of the siRNA to the Cs nanoparticles was observed due to the presence of strong brilliant bands, which suggested that the Cs polymer strongly protected the loaded siRNA (Figure 1A). However, for the adsorbed method, a weak band was obtained, which indicates the weak interaction between the Cs and siRNA (Figure 1A).

### The synthesized cs NPs protect their payload from nuclease degradation

The ability of the synthesized NPs to protect their payload was assessed. A serum protection test was carried out for Cs nanoparticles in 10% canine serum. Figure 1B shows strong and bright bands of the siRNA loaded by the entrapment method even after 2 weeks of incubation with serum. In contrast, compared with the entrapped siRNA, the CsDHA-TPP NPs partially protected the surface of the siRNA, as a less dense band was observed. Naked siRNA started to degrade as early as 30 min, and most of the siRNA was degraded after 24 h of incubation (Figure 1B). This degradation is due to the mixing of siRNA with serum and freezing steps. Due to the high encapsulation effect of the NPs loaded via the entrapment method in addition to the strong ability of the loaded siRNA to protect against degradation in the serum, these NPs were used in subsequent experiments.

**Figure 2.**
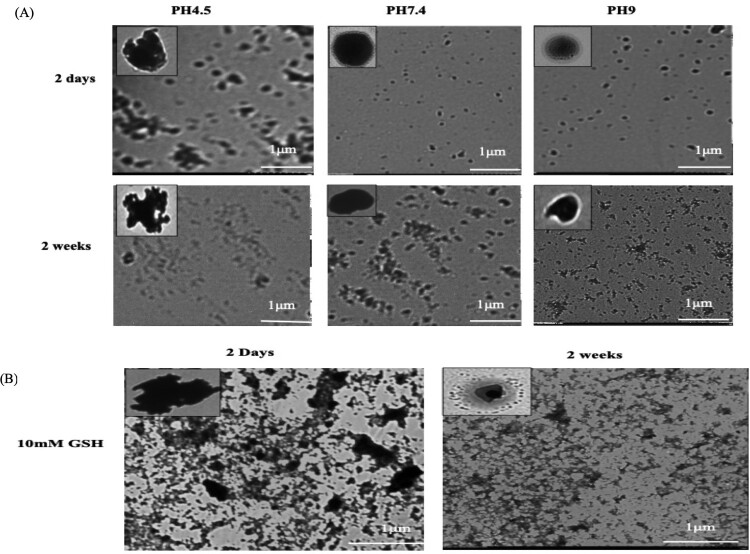
TEM images of the morphological changes in the Cs NPs under different pH and GSH conditions. (A) Morphological changes in the Cs NPs at various pH values (4.7, 7, and 9). (B) Changes in the morphology of Cs NPs treated with 10 mM GSH.

The *in vitro* release profiles of DHA and siRNA-loaded Cs-TPP nanoparticles were investigated for 14 days in PBS at pH 7.4, 9, 4.5, and 10 mM GSH to mimic the normal environment of blood, endosomes, and infected RBCs, respectively. The results showed that the lowest initial release of siRNA at neutral and alkaline pH values occurred in the first two days. For acidic pH, a greater initial release percentage started after 4 days of incubation, followed by a greater cumulative release of siRNA from the Cs nanoparticles. However, a very low percentage of DHA was released even after 14 days of incubation (Figure 1C). In addition, the release profile of the loaded siRNA was studied under the effect of GSH, and the results showed a greater initial release of siRNA as early as 30 min of incubation with continuous high release for up to 4 days of incubation, followed by a lower release profile until 14 days of incubation (Figure 1C). These results were confirmed by the morphological changes in the incubated nanoparticles, as shown by the TEM images. The images showed severe destruction and irregular shapes of the outer membrane of the nanoparticles under the effects of acidic pH (Figure 2A) and GSH (Figure 2B). This irregularity was severe in the GSH-incubated NPs and started as early as 2 days of incubation in comparison with less severe irregularity under the effect of acidic pH after 2 days of incubation and increased with increasing incubation period. Moreover, for NPs incubated under neutral pH, the NP outer membrane still maintained its regular smooth shape with slight aggregation after 14 days (Figure 2A).

### CsDHA NPs are safe for RBCs and mammalian cells

To investigate the hemolytic effect of CsDHA NPs on RBCs, a hemolysis assay was performed. None of the tested concentrations had a hemolytic effect on the RBCs (Figure S3A). Moreover, *in vitro* treatment of Madin-Darby bovine kidney (MDBK) cells with different concentrations of synthesized NPs resulted in no cytotoxic effects until 50 µg/mL was reached, Additionally, to exclude the antiparasitic effect of the CsDHA NPs, different concentrations of the synthesized CsDHA NPs were tested for their effects on the *in vitro* growth of *B. gibsoni,* and the results showed that the Cs NPs significantly inhibited parasite growth starting at 12.5 µg/mL, with an inhibition rate higher than 60% (Figure S3B). On the basis of these results, all the prepared CsDHA NPs used for further experiments were prepared at a concentration of 10 µg/mL.

The selectivity index (SI) is a ratio that measures the window between cytotoxicity and antiprotozoal activity by dividing the given IC_50_ value into the CC_50_ value (IC_50_/ CC_50_). The higher the SI ratio, the theoretically more effective and safe a drug would be during in vivo treatment for a given protozoal infection [20]. In the present study, the selectivity index for the empty CsDHA NPs was higher than 4. This result confirm that the CsDHA NPs is safe for the application on mammalian cells.

### Rates of successful targeting and increased internalization of CsDHA NPs by *B. gibsoni* iRBCs

**Figure 3.**
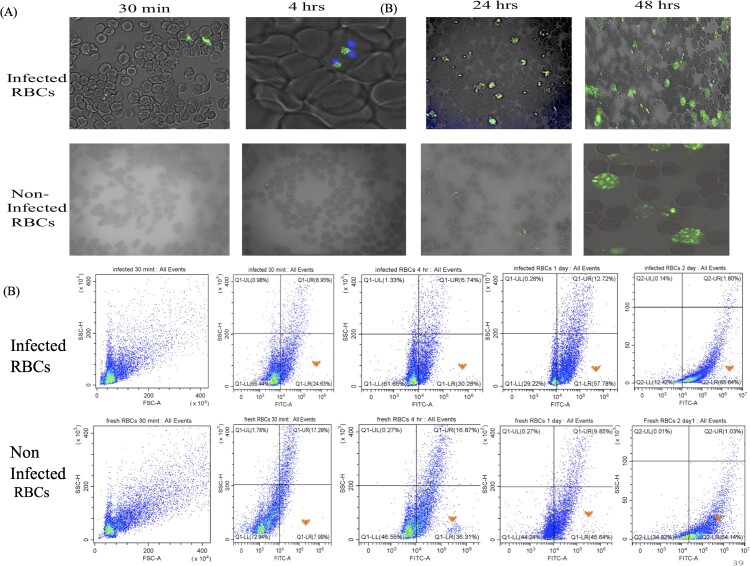
Cellular internalization of Cs NPs by *B. gibsoni*-infected and uninfected RBCs. (A) Fluorescence laser microscopy images of internalized Fit C-labelled Cs NPs inside infected and noninfected canine RBCs. (B) Time-dependent internalization rate of Fit C-labelled Cs NPs inside infected and noninfected canine RBCs.

**Figure 4.**
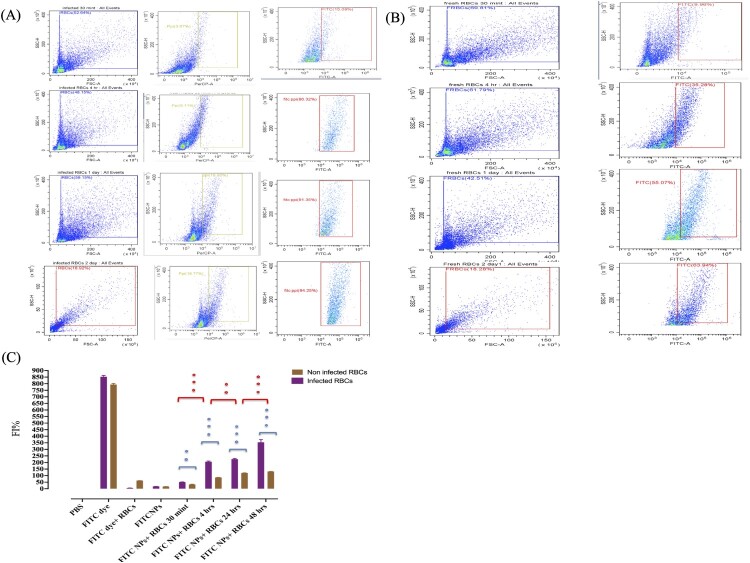
Targeting ability of the Cs NPs to *B. gibsoni*-infected and noninfected RBCs. (A and B) Flow cytometry quantitative analysis of internalized Fit C-labelled Cs NPs inside infected and noninfected canine RBCs. (C) Fluorescence intensity of Fit C-labelled Cs NPs inside *B. gibsoni*-infected and uninfected RBCs. P<0.05, p<0.001, p <0.0001

In the present study, the affinity of CsDHA NPs for *B. gibsoni* iRBCs was evaluated. To detect the nanoparticles, the Cs DHA NPs were first cross-linked with FITC dye to yield FITC labelled CsDHA NPs. Laser scanning microscopy (LSM) revealed that the CsDHA NPs were able to bind specifically to iRBCs rather than to uninfected RBCs, with a significant increase (*P*<0.05) in the percentage of internalized FITC labelled CsDHA NPs inside the iRBCs with increasing incubation time (Figure 3A). These results were confirmed using flow cytometry, which confirmed that the internalization of FITC labelled CsDHA NPs inside the iRBCs was 85%, while that of the noniRBCs was 64% (Figure 3B, 4A, and 4B). Furthermore, the fluorescence intensity of the FITC labelled CsDHA NPs was greater in the iRBCs than in the uninfected RBCs (Figure 4C).

Importantly, we assessed whether *B. gibsoni* can process siRNA via the canonical pathway. CsDHA NPs loaded with siRNA NFT and naked siRNA NF were incubated with *B. gibsoni* iRBCs. Neither CsDHA NPs loaded with siRNA NFT nor naked siRNA NFT were able to inhibit *B. gibsoni* growth *in vitro,* with inhibition rates of only 10.87 and 18.88%, respectively (Figure S4A). Moreover, the siRNAs either loaded on CsDHA NPs or naked were not able to silence the nitrate transporter, as indicated by the qPCR results (Figure S4B).

### ssRNA-hAgo2 complexes were successfully assembled

**Figure 5.**
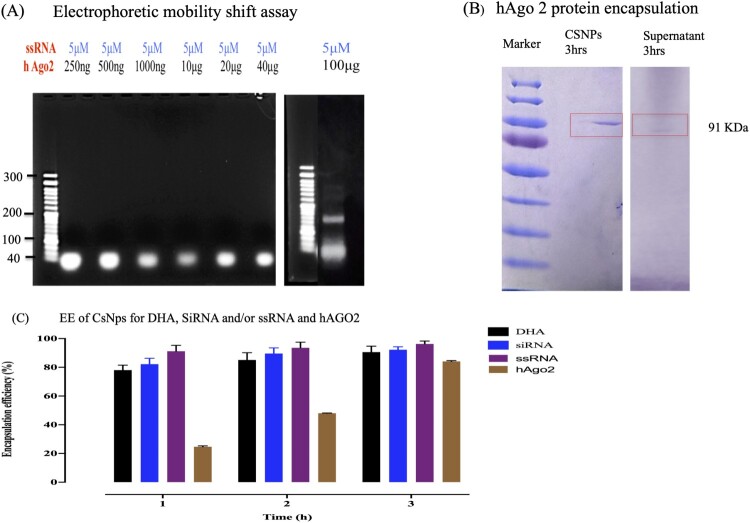
ssRNA-hAgo2 complex assembly. (A) Complex assembly confirmation by electrophoretic mobility shift assay (EMSA). (B) Encapsulation efficiency of Cs NPs for hAgo2 by SDS‒PAGE. (C) Encapsulation efficiency of Cs NPs for DHA, ssRNA, siRNA, and hAgo2.

We demonstrated the formation of complexes by EMSA. In this assay, the interaction between the RNA and protein is stabilized. Different concentrations of hAgo2 were incubated with 5 μM ssRNA. Only samples incubated with 100 μg of hAgo2 exhibited retardation of ssRNA, and unbound RNA migrated faster (Figure 5A). Therefore, complex formation was observed by the appearance of approximately 50-nucleotide bands compared with bands of approximately 21 nucleotides corresponding to unbound ssRNA. After successful assembly between ssRNA and siRNA with hAgo2 protein, the assembled complexes were loaded onto CsDHA NPs. Then, the entrapment ability was assessed for DHA, ssRNA, siRNA, and hAgo2 with 90, 96, 92, and 84% EE after 3 hrs of incubation, respectively (Figure 5C). Moreover, the EE of the hAgo2 protein was confirmed by SDS‒PAGE (Figure 5B).

### Cs DHA NPs introduced proteins into *B. gibsoni* iRBCs

**Figure 6.**
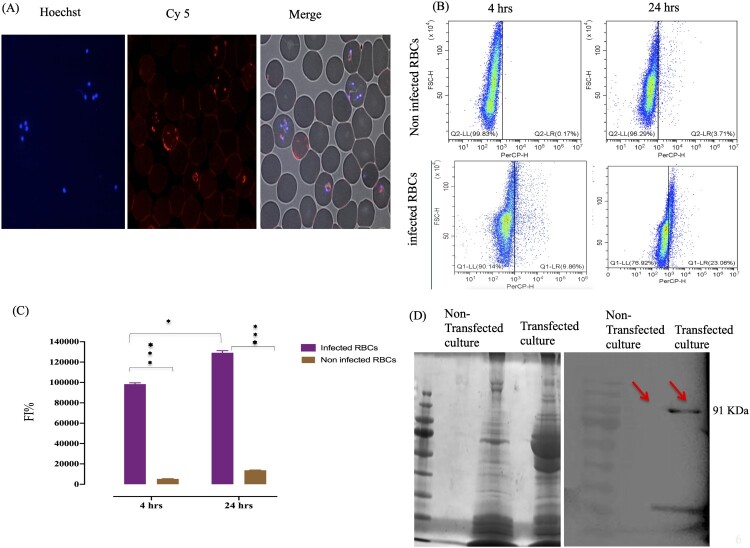
Assembled complex transfection of *B. gibsoni* iRBCs. (A) Fluorescence laser microscopy images of B. gibsoni-infected RBCs transfected with Cy5-labelled ssRNA. (B) Flow cytometry quantitative analysis of *B. gibsoni*-infected and noninfected canine RBCs transfected with CY5-labelled ssRNA. (C) Fluorescence intensity of *B. gibsoni*-infected and noninfected canine RBCs transfected with CY5-labelled ssRNA. (D) SDS‒PAGE and western blotting of B. gibsoni-infected RBCs transfected with the tagged hAgo 2 protein. P<0.05, p<0.001. p <0.0001.

We initially transfected iRBCs with the assembled complex using CsDHA NPs loaded with His-tagged hAgo2+Cya5-labelled ssRNA. We detected Cya5-labelled ssRNA only in parasites treated with complexes, as confirmed by laser scanning microscopy (Figure 6A). No transfection was observed in noninfected RBCs. Moreover, flow cytometric analysis with fluorescently labelled ssRNA indicated that 9.8%–23% of iRBCs were transfected after 4–24 hrs, while 3.7% of noninfected RBCs exhibited greater fluorescence intensity in iRBCs than in noniRBCs, as confirmed by the FI results (Figure 6B and C). To demonstrate hAgo2 protein transfection, His-tag–hAgo2 was transfected into iRBCs, and parasite lysates were prepared and subjected to SDS‒PAGE and western blotting with an anti-His tag antibody. A band corresponding to the recombinant hAgo2 protein was detected in the parasite lysate of the transfected culture rather than in that of the nontransfected culture (Figure 6D).

### The assembled complexes induce gene silencing and significantly inhibit *B. gibsoni* growth *in vitro*

**Figure 7.**
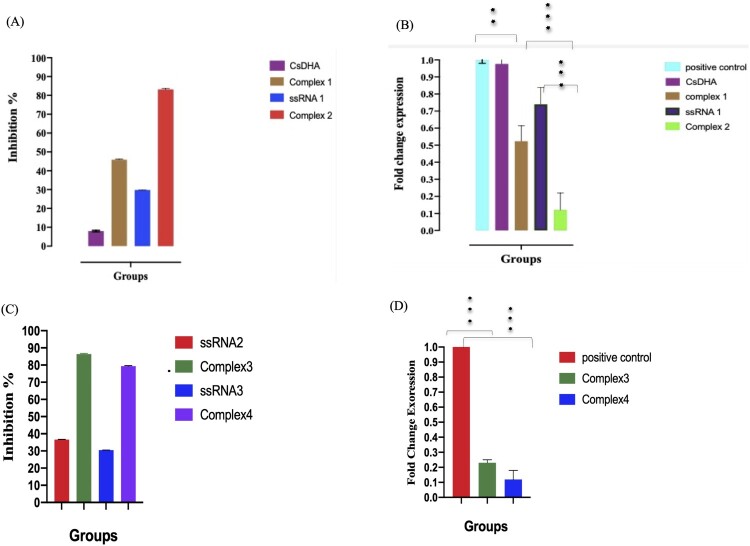
*In vitro* inhibitory effect of Cs DHA NPs with assembled complexes on *B. gibsoni* growth. (A) *In vitro* inhibition rate of both hAgo2 +siRNA NFT- and hAgo2 +ssRNA NFT-loaded Cs NPs against *B. gibsoni* growth. (B) Fold changes in NFT expression after treatment with both hAgo2 +siRNA NFT and hAgo2 +ssRNA NFT-loaded Cs NPs. (C) *In vitro* inhibition rates of both hAgo2 +ssRNA v-type ATPase- and hAgo2 +ssRNA p-type ATPase-loaded Cs NPs against *B. gibsoni* growth. (D) Fold changes in v-type ATPase and p-type ATPase expression after treatment with hAgo2 +ssRNA v-type ATPase and hAgo2 +ssRNA p-type ATPase-loaded Cs NPs. P< 0.05, p < 0.001. p < 0.0001.

ssRNA 1 and Cs NPs complexes 1 and 2 were incubated with *B. gibsoni* iRBCs for 4 days at 37°C, and on the 4^th^ day, the iRBCs were collected, and thin blood smears were prepared, stained with Giemsa stain and examined using a light microscope. The results showed that Cs NPs complexes 1 and 2 significantly inhibited (*P*<0.05) *B. gibsoni* growth *in vitro,* with inhibition rates of 48.3 and 83.2%, respectively, while ssRNA 1 inhibited *B. gibsoni* growth by only 28.5% (Figure 7A). The hAgo2 + ssRNA complex exhibited a greater inhibitory effect on *B. gibsoni* growth and NFT expression than did the hAgo 2+ siRNA complex (Figure 7A). These results were confirmed by the RT‒PCR results, in which complex 2 had the greatest silencing effect on NFT, followed by complex 1 and ssRNA1 (Figure 7B). To confirm the ability of hAGO protein to manipulate RNAi processing, two additional complexes against *B. gibsoni* V-type ATPase and P-type ATPase transporters were transfected into *B. gibsoni*-infected RBCs, and their inhibitory and silencing effects were tested. As expected, both complexes showed greater inhibitory effects and downregulated the expression of the targeted transporters (Figure 7C and D).

### NFT silencing inhibits lactate metabolite efflux from iRBCs

**Figure 8.**
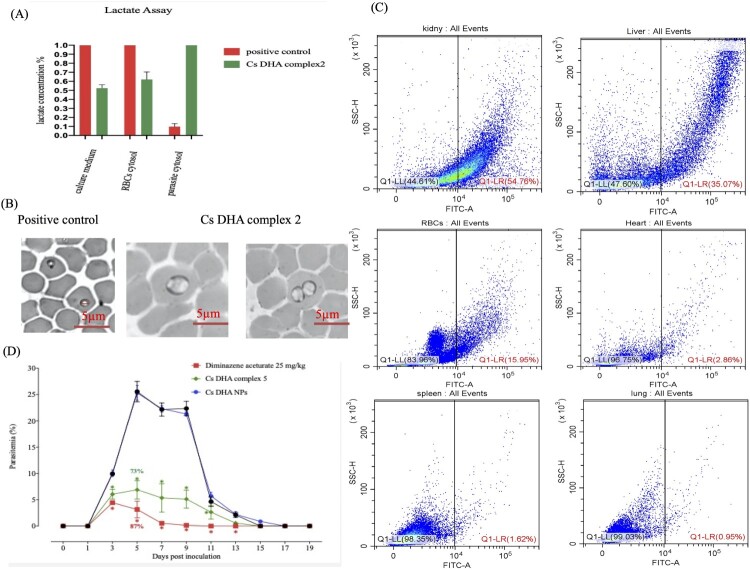
Lactate assay, *in vivo* biodistribution and inhibition of Cs NP complex 5. (A) Different lactate concentrations before and after the silencing of NFT. (B) Changes in the morphology and size of *B. gibsoni* parasites before and after the silencing of NFT. (C) Flow cytometry quantitative analysis of Fit C-labelled Cs NPs in the kidney, liver, red blood cell (RBC), heart, lung, and spleen. (D) *In vivo* inhibitory effect of assembling CsDHA complex 5 on *B. microti* growth in mice.

To assess the function of NFT in the efflux of lactate metabolites, we measured the concentrations of the lactate formed inside and outside the parasite cytosol and RBC cytosol. The results showed that the concentration of lactate inside the parasite cytosol was significantly greater (*P*<0.05) in the treated group than in the positive control group that did not receive treatment. The lactate concentration in the RBC cytosol was significantly lower (*P*<0.05) in the treated group than in the positive control group. The lactate concentration in the culture medium was significantly greater (*P*<0.05) in the untreated group than in the treated group (Figure 8A). Moreover, the accumulation of lactate metabolites resulted in swelling of the parasite cytosol, as shown in Figure 8B.

Furthermore, we determined the *in vivo* distribution of CsDHA NPs in different *B. microti*-infected mouse tissue; the concentration of CsDHA NPs in blood and various tissues was measured by flow cytometry. Twenty-four hours after injection, the kidney and liver were preferential sites for Cs NPs accumulation, as expected, at 54.76% and 35.07%, respectively (Figure 8C). However, in other organs, including the heart, lung, and spleen, a nonsignificant accumulation of Cs NPs was observed, with accumulations of 2.86, 1.62, and 0.95%, respectively (Figure 8C). Interestingly, the amount of accumulated Cs NPs in the blood (15.95%) was significantly greater than that in the lung, spleen, and heart, which indicates the successful ability of the synthesized NPs to target infected RBCs, as confirmed before *in vitro* studies, and this targeting ability might be due to the high expression of the DHA ligand loaded with GLUT on the iRBC surface.

Importantly, compared to control mice, *B. microti*-infected mice treated with CsDHA complex 5 showed significant inhibition (*P*<0.05) of parasitemia from days 3–11 posttreatment, which was nearly similar to the effect of the administered control drug, DA (Figure 8D). CsDHA complex 5 caused 73% inhibition of parasite growth during the day, with peak parasitemia (day 5) (Figure 8D). The obtained results confirmed for the first time the promising antibacterial efficacy of CsDHA complex 5.

## Discussion

RNA interference is a process in which most eukaryotic cells use RNA to silence genes [21]. The biology of numerous parasites and diseases has been extensively studied using this technique [21]. Unfortunately, the primary constraint of this approach is that certain organisms lack RISC enzymes, making siRNA impractical [22]. A previous study demonstrated that *Babesia* lacks RISC machinery [23]. Therefore, the RISC-dependent silencing method cannot be used to study gene function in this organism. Importantly, some studies have developed a novel alternative method for silencing genes in *Cryptosporidium* and *Plasmodium*, which are also parasites that lack the RISC machinery [24,25]. These alternative methods depend on noncanonical RNAi pathways that require only the AGO2 protein [26]. However, the effective delivery of naked RNAi payloads to the required target site without affecting its biological efficacy is a key advantage. Following this pattern, in this study, we developed a Cs NPs gene delivery system targeting *B. gibsoni* iRBCs. Chitosan was successfully extracted from shrimp shells with moderate DP% and DD%, and these rates effectively achieved the ability of the synthesized NPs to undergo endosomal scaping and overcome opsonization. This may be attributed to the protonation ability of the extracted chitosan, which can absorb water from outside the endosome into inside the endosome, resulting in swelling and rupture and subsequently leading to the release of the entrapped NPs to reach their target without being removed by endocytosis. In addition, the ionic gelation method used for the synthesis of Cs NPs resulted in the production of small and assorted spherical NPs with regular shapes. This small particle size is due to the strong ionic interactions between the negatively charged siRNA and the positively charged chitosan polymer. While, the spherical shape of the synthesized NPs is related to the excellent cross-linking ability of TPP, which successfully neutralized the CsNH3+ groups. Our results were in accordance with previous studies [12,27,28]. Taken together, this cross-linking ability results in the formation of a strong Cs polymer, which can protect the loaded DHA and siRNA from nuclease degradation with a constant release rate of its payloads even after 14 days of incubation at neutral and alkaline pH. In contrast, under acidic pH and GSH conditions, the release profile of the loaded substances increased due to the swelling of the Cs NPs. This swelling is attributed to the protonation of the amine group of the Cs NPs in the acidic medium, resulting in the absorption of water from the surrounding environment. Such absorption leading to weak crosslinking of the Cs and TPP cross-linker and poor control of diffusion-based release. These leading to increase the release of the loaded siRNA. However, a very low percentage of DHA was released even after 14 days. This is attributed to the fact that siRNA is bound near the particle surface, and subsequently, siRNA first diffuses to the surrounding medium, followed by the release of DHA, which is entrapped in the core of the NPs [29]. This pattern of the payloads released under acidic pH conditions only confirmed the ability of the synthesized NPs to protect the loaded siRNA during its passage through the body until it reached the targeted RBC site. After confirming the stability of the synthesized Cs NPs, we studied the ability of the synthesized Cs NPs to selectively target *B. gibsoni* iRBCs. The results revealed greater internalization of FIT C-labelled Cs NPs by iRBCs than by noniRBCs. This selective targeting is attributed to the loaded DHA, which is a ligand of GLUT that is expressed on the surface of RBCs. The higher internalization in the iRBCs than in the noniRBCs may be due to GLUT being expressed at 40% greater levels on the surface of the iRBCs than on the surface of the noniRBCs to meet the higher requirement of the parasite for glucose to fuel its energy requirements [30]. After that, we studied the ability of Cs NPs to deliver siRNA and to induce silencing. Since CsDHA NPs can traverse directly into the iRBC and bind the parasite, they might improve the inhibitory activity of RNA–nanoparticle conjugates. Both CsDHA NPs loaded with siRNA NFT and naked siRNA NFs were incubated with *B. gibsoni* iRBCs, and the results showed that neither CsDHA NPs loaded with siRNA NFT nor naked siRNA NFT were able to inhibit *B. gibsoni* growth *in vitro* or to silence the nitrate-formatted transporter, as indicated by the qPCR results. These nonsignificant results may be attributed to the fact that *B. gibsoni* lacks the canonical pathway for siRNA processing; hence, siRNA application in this parasite is impractical [23]. These results are not in accordance with those of [31], who reported that dsRNA against DNA gyrase subunits A and B, the DNA-directed RNA polymerase beta subunit (rpo B), and the thiostrepton interaction site (ribosomal L11 protein) can inhibit the *in vitro* growth of *Babesia bovis*. The authors attributed this result to the silencing of a part of the gene by RNAi leading to the destruction of the whole gene transcript. After the nonsignificant results obtained by CsDHA NPS siRNA against NFT, we wanted to study the Dicer nondependent pathway in enhancing siRNA and ssRNA processing in *B. gibsoni*. In this pathway, the processing of siRNA or ssRNA depends only on the Ago 2 protein, so ssRNA and siRNA +hAgo2 assembly was performed. The successful assembly of the complex obtained may be because ssRNA-Ago2 complexes can be assembled *in vitro* and retain slicer activity [13,32]. Furthermore, the transfection of the assembled complex into iRBCs was confirmed, and the results confirmed the successful transfection of the complexes, with greater internalization in iRBCs than in noniRBCs. This significant difference in the transfection rate between the iRBCs and noniRBCs is attributed to the ability of the CsDHA NPs to target the highly expressed GLUT on the iRBCs, as mentioned before. Next, we studied the ability of the assembled complexes to induce gene silencing. The assembled complexes significantly inhibited *B. gibsoni* growth *in vitro.* The hAgo2 + ssRNA complex exhibited a greater inhibitory effect on *B. gibsoni* growth and NFT expression than did the hAgo 2+ siRNA complex. Such great inhibitory effect possibly because hAgo2 significantly binds and produces a slicing effect on ssRNA compared with siRNA. Moreover, the loading of hAgo 2 protein to the CsNPs enhances the nondicer-dependent pathway of siRNA and ssRNA processing and results in successful silencing of NFT, as shown by the RT‒PCR results.

After the significant silencing effect of the loaded hAgo 2 protein+ ssRNA, we wanted to confirm the ability of this complex to manipulate the nondicer-dependent pathway in *B. gibsoni.‏* Two additional Ago2+ssRNA complexes against p-type ATPase and V-type ATPase also inhibited *B. gibsoni* growth *in vitro* via downregulation of targeted transporter expression. This strong inhibitory effect indicated that the loading of hAgo 2 protein into the Cs NPs significantly enhanced the inhibitory effect of siRNA and ssRNA on *B. gibsoni* growth *in vitro*. Our results were in accordance with those of [26], who successfully silenced gene expression in *Cryptosporidium* using preassembled complexes of hAgo2 and ssRNA.

To confirm the silencing effect of the formed complex on the function of the targeted transporter, we studied the effect of CsDHA complex 2 on the function of NFT. NFT is expressed on the parasite plasma membrane after invading RBCs to help the parasite eliminate lactate (the end product of the anaerobic glycolysis pathway). *B. gibsoni* usually depends on anaerobic glycolysis to fuel its high energy demand. As a result of this pathway, lactate is formed and accumulates inside the parasite cytosol, and the parasite has to efflux this lactate from its cytosol to the RBC cytosol to eliminate the associated toxicity if lactate accumulates inside its cytosol. As a result of NFT efflux, lactate is released from the parasite cytosol to the RBC cytosol. After that, lactate is released from the RBC cytosol to the culture medium through monocarboxylate transporter 1 (MCT1), which is normally expressed on the RBC membrane. The results confirmed the accumulation of lactate inside the parasite cytosol in the treated group compared with the nontreated group. This result indicates that NFT function in the treated group was downregulated due to the silencing effect of CsDHA complex 2 on NFT. Furthermore, the higher lactate concentration in the culture medium of the nontreated group indicated the specificity of the formed complex in targeting NFT without affecting MCT1 function. Finally, we investigated the *in vivo* biodistribution of the i/v injected CsDHA NPs in different mouse organs. The highest accumulation rate of CsDHA NPs was detected in the kidney and liver, as expected, due to the clearance effect of the body, followed by that of RBCs. However, there was no significant accumulation of NPs in the other organs collected, which may be attributed to the selective targeting of DHA to overexpress GLUT on the RBC membrane, as explained previously. However, this study investigated the cytotoxicity assay of Cs NPs using MDBK, future studies confirming the obtained results using MDCK are required. In the present study, we evaluated the silencing of the selected targeted transporters by measuring the reduction in messenger RNA (mRNA) by qPCR. However, further studies are required to clone and to express the selected proteins from treated and nontreated culture as an initial step. Then, perform quantitative detection of these target proteins using Western blotting.

## Conclusion

In the present study, we developed a novel nanodelivery system to target *B. gibsoni*-infected RBCs without nonspecific binding or lysing of RBCs. Cs nanoparticles likely interact with GLUT1 expressed on the iRBC surface. Through this synergistic effect of selective targeting, CsDHA NPs can effectively deliver gene therapies to iRBCs with high accumulation and direct contact of the loaded therapies on the parasite. With this delivery system, we were able to deliver gene therapy for the first time to *Babesia* parasites with novel silenced gene expression using preassembled complexes of hAgo2 and ssRNA, which overcomes the previous impractical siRNA silencing process in *Babesia* species due to the absence of the canonical pathway of siRNA processing in this parasite. The findings obtained might also be useful for other organisms with deficient or absent siRNA pathways. Because of the targeting specificity of the synthesized NPs to GLUT, which is expressed on all mammalian RBCs, this delivery system can be used for all haemoprotozoan parasites. This method may prove to be a new tool for identifying gene functions and drug targets for treating babesiosis and other haemoprotozoan parasites and attenuating parasites for live vaccines. These findings might herald a paradigm shift in the field of antibiosis toward the use of chitosan nanoparticles as multipurpose antibabesiosis agents.

## List of abbreviation

**siRNAs**: Small interfering RNAs

**RISC**: RNA-induced silencing complex enzymes

**hAgo2**: Human enzyme argonaute 2

**ssRNA**: Single-stranded RNA

***B. gibsoni*** *: Babesia gibsoni*

***B. canis*** *: Babesia canis*

**iRBCs**: Infected red blood cell

**Cs**: Chitosan

**GLUT-1**: Glucose transporter

***Pf*:** *Plasmodium falciparum*

**DHA**: Dehydroascorbic acid

***B. microti*** *: Babesia microti*

**DP**: Deproteinization

**DD**: Deacetylation degree

**NFT**; Nitrite format transporter

**EMSA**: Electrophoretic mobility shift assay

**TEM**: Transmission electron microscopy

**LSM**: Laser scanning microscopy

**FITC**: Fluorescein isothiocyanate

**PBS**: Phosphate-buffered saline

**qPCR**: Quantitative polymerase chain reaction

**GSH**: Reduced glutathione

**SDS‒PAGE**: Sodium dodecyl sulphate poly acrylamide gel electrophoresis

**MDBK:** Madin-Darby bovine kidney cells

**MCT1**: Monocarboxylate transporter 1

**NP:** Nanoparticle

**TPP**: Tripolyphosphate

## Author contributions

Conceptualization: Shimaa Abd El-Salam El-Sayed, Xuenan Xuan. Data curation: Shimaa Abd El-Salam El-Sayed, Mohamed Abdo Rizk. Formal analysis: Shimaa Abd El-Salam El-Sayed. Investigation: Shimaa Abd El-Salam El-Sayed, Xuenan Xuan Methodology: Shimaa Abd El-Salam El-Sayed, Hang Li, Iqra Zafar, Project administration: Xuenan Xuan. Resources: Mohamed Abdo Rizk, Shimaa Abd El-Salam El-Sayed, Xuenan Xuan. Software: Mohamed Abdo Rizk, Uday Kumar Mohanta, Shengwei Ji, Ma Zhuowei, Thom Do, Yongchang Li, Jerzy Jaroszewski. Supervision: Xuenan Xuan. Validation: Shimaa Abd El-Salam El-Sayed. Visualization: Mohamed Abdo Rizk, Shimaa Abd El-Salam El-Sayed. Writing – original draft: Shimaa Abd El-Salam El-Sayed, Mohamed Abdo Rizk. Review & editing revised manuscript: Jerzy Jaroszewski. Writing – review & editing: All authors.

## Ethics approval and consent to participate

All the experimental protocols used in this study were approved by the Animal Care and Use Committee of Obihiro University of Agriculture and Veterinary Medicine (Approval Nos. 22–23). The pathogen experiment IDs were as follows: animal experiment: 20-280; pathogen experiment: 201712-5; DNA experiment: 629-2; and *Babesia Microti*: 20170905.

## Supplementary Material

Supplementary materials 1.pdf

Table S1.pdf

## Data Availability

On reasonable request, the corresponding author will provide the datasets created and/or analysed during the current work.
