# Supplementary material for: Preassembled complexes of hAgo2 and ssRNA delivered by nanoparticles: a novel silencing gene expression approach overcoming the absence of the canonical pathway of siRNA processing in the apicomplexan parasite Babesia microti, blood parasite of veterinary and zoonotic importance
Source: Emerg Microbes Infect. 2024 Dec 9;14(1):2438658. doi: 10.1080/22221751.2024.2438658 (PMC11721618; doi:10.1080/22221751.2024.2438658)
Supplement: Supplementary materials 1.pdf [file TEMI_A_2438658_SM1975.pdf]

Supplementary materials 1: Full description of some experiments used in this study

Materials and methods

Chitosan production and characterization of the extracted chitosan

Chitosan extraction was performed according to [1]. Shrimp shells (300gm) were dried in the sun for 2 days. After sun drying, the shells were ground into powder. Dried Powder shrimp shells were placed in opaque plastic bottles and stored at ambient temperature. Then, the shrimp shells were demineralized for 24 hours at room temperature using 5% HCL at a ratio of 1:6 (w/v). The demineralized shells were rinsed with water to get rid of the acid and calcium chloride, and dried at 60°C. The demineralized shells were soaked in 5%NaOH solution with a 1:10 (w/v) solid-to-solvent ratio for 48 hours at 60–70°C. Following processing, distilled water was used to remove the excess NaOH from the residue followed by drying at 60°C. In this stage, the obtained substance is known as chitin. Yield: weight of chitin 22.75 gm. After that, the obtained chitin was deacetylated to chitosan by removal of the acetyl with a 60% NaOH solution. The solution was heated for 2 hours for deacetylation. After rinsing with distilled water and drying at 60ºC, the deacetylated chitin (now known as chitosan) was ready for use. Yield: weight of chitosan: 15.84 gm. After that, the extracted chitosan was characterized using De acetylation degree calculated by Potentiometer Titration. Briefly, 20 mL of 0.3 N hydrochloric acid was used to dissolve 0.5 grams of chitosan. This solution was titrated with a 1 N NaOH solution following the addition of 400 mL of distilled water. A pH value vs. NaOH titration volume curve was produced. For every highlighted transition, the inflection points of the curve were determined. The following equation was used using the volume of NaOH at each inflection point:

$NH_2\% = 16.1 \times (y - x)/M$ (2); where M is the weight of chitosan used, x is the first inflection point on the graph of measured pH vs. titration volume, and y is the second inflection point.

Free $NH_2\%$ or DD% $= NH_2\% / 9.94\% \times 100\%$; where 9.94 % is the theoretical NH_2 percentage.

Chitosan theoretic NH_2 content % = (16/161) × 100% = 9.94%

0.016: equal to NH_2 content (g) in 1 mL of 1 M HCl.

Entrapment efficiency

The entrapment efficiency was calculated using the following formula:

$$\text{Entrapment efficiency\%}\ \ C_{sample} - C_{supernatant} / C_{sample} \times 100$$

where C_{sample} is the concentration of DHA, siRNA,ssRNA, and hAgo2 added and $C_{supernatant}$ is the concentration of loaded substances in the supernatant [2]. All measurements were performed in triplicate, and data are reported as mean ± standard deviation. Moreover, the encapsulation of hAgo2 protein was confirmed by SDS gel electrophoresis for loaded NPs and the remaining supernatant after 3 hours of loading.

***In vitro* release studies and determination of the hemolytic and cytotoxic effects of the developed NPs**

Samples (4 mL) were centrifuged at 10,000 r.p.m for 30 min at 37°C and pellets were re-suspended in 3 mL of PBS (pH 7.4, 9,and 6.4) and GSH (10 mM) solutions. The re-suspended pellets were incubated in a shaker at 37°C/150 rpm/8

days. At different time intervals, the samples were centrifuged at 10,000 rpm for 30 min at 25°C. The supernatant recovered from centrifugation was used for further analysis, and the pellets were kept for morphological changes with TEM. An equivalent volume of fresh PBS and GSH was added to the sample. The amount of siRNA present in the supernatant was analyzed using a UV-vis spectrophotometer (Ultraspec 2100 pro Amersham Biosciences) at 260 nm.

Hemolysis assay was done according to the method described by [3]. 200µl non-infected RBCs were suspended in 10 ml PBS 1X to prepare 2%HCT. 100 µl were incubated with 100 µl of different concentrations of CsDHA-TPP NPs (50 µg/ml, 25 µg/ml, 12.5 µg/ml, 6.25 µg/ml, 1 µg/ml) in triplicate. The samples were incubated for 1hr at 37c and the released Hb was measured by UV-vis spectrophotometer (Shimadzu UV-1800, Shimadzu Scientific Instruments, Japan) at 540 nm.

The cytotoxic assay was performed as follows; 5×10^4 cells/ml of Madin-Darby Bovine Kidney (MDBK) cells were seeded on 100 µl per well in a 96-well-cell culture plate and incubated for 24 h. One hundred microliters of 2-fold NP dilutions were added to each well to a final concentration of (50 µg/ml, 25 µg/ml, 12.5 µg/ml, 6.25 µg/ml, and 1 µg/ml) in triplicates. After 24 h, 10 µl of CCK-8 was added to each exposed NP. After 4 h incubation, the absorbance values were determined at 450 nm using an MTP-500 microplate reader (Multi Skan Sky High, TC, Japan). The wells with only the culture medium were used as blank, while those containing cells in a medium with 1% acetic acid were used as control.

Cellular internalization

Living *B .gibsoni* cultures (3% parasitemia, 5%HTC) were incubated in complete RPMI at 37 °C with gentle stirring in the presence of 10 µg/ml of FITC-

DHA-siRNA-encapsulated Cs NPs for (30 min 4hrs, 24hrs, 48hrs) protected from light. The infected RBCs were then thoroughly washed three times with PBS (1500 r.p.m/5 min). Hoechst 33342 nuclear stain was added at a final concentration of 2 μg/ml and cells were further incubated for another 10 min. cells were washed twice with RPMI, then diluted 20 times in RPMI to reach 0.15% hematocrit. Finally, 10 μL of this mixture was put on a glass slide, covered with a glass cover. Non-infected RBCs (treated and not treated with CsDHA-siRNA NPs) and iRBCs not treated with CsDHA-siRNA NPs were used as a control. Preparations were then analyzed by all-in-one laser scanning microscopy. For flow cytometry, the infected RBCs were stained with propidium iodide (PI) nuclear stain at a final concentration of 2 μg/ml and cells were further incubated for another 10 min. Then, the cells were washed with PBS twice and fixed with paraformamide- PBS 4%. The preparations were then analyzed using flow cytometer (Beckman Coulter, life science). The single-cell population was selected on a forward-side scatter scattergram. FITC was excited at (488nm), and its fluorescence collected through a 525/40BP. While, PI was excited with (488nm) and its fluorescence was collected using a (690/50BP). After that, to confirm the time-dependent uptake of CSDHA-TPP NP by RBCs, *B. gibsoni* infected (1% parasitemia, 5%HTC, 100μl total volume) and noninfected RBCs were exposed to 10 μg/ml of FITC-DHA-siRNA-encapsulated Cs NPs for 30 min, 4hrs, 24hrs, 48hrs at 37°C. Cs NP content inside cells was determined in RBC lysates after FITC-Cs NPs exposure, as previously described by [4]. After treatment, iRBCs were washed three times with PBS (pH 5.0) and once with 5 mM EDTA (pH 5.0) to remove nanoparticles those not taken up by the RBCs, including those attached to the cell membrane and the plastic of the well. The RBCs were then disrupted with 100μl lysis buffer (pH 8.0), containing 2% SDS and 50 mM EDTA. Non-infected RBCs

exposed to the vehicle (PBS; pH 7.4), are served as a control for intrinsic fluorescence. Wells without cells, but incubated with Cs NPs and processed as just described are served as the control for residual fluorescence after washing. iRBCs, and non-IBCs with FITC dye are also used as control. Fluorescence in RBC lysates was immediately measured in a fluorescence multi-plate reader (Spectra max iD5, Molecular devices) at 458 nm (excitation) and 538 nm (emission).

Quantitative Polymerase Chain Reaction (qPCR) and Lactate assays

The following conditions were used: 50°C for 2 minutes, 95°C for 2 minutes, and 40 cycles at 95°C for 15 seconds 60°C for 15 seconds, and 72°C for I minute, using the (Real time PCR system, Quant studio 3, Applied biosystem). We evaluated the relative fold change by the delta-delta method (ratio = $2^{\Delta Ct\ sample - \Delta Ct\ control}$), which compares the cycle threshold (Ct) values of treated samples and un transfected parasites. For controls and reference genes, we used the *B. gibsoni Beta-actin*. All samples were analyzed in triplicate in at least 2 independent experiments.

References

1. El-Araby A, El Ghadraoui L, Errachidi F: **Physicochemical Properties and Functional Characteristics of Ecologically Extracted Shrimp Chitosans with Different Organic Acids during Demineralization Step**. *Molecules* 2022, **27**(23).
2. Katas H, Alpar HO: **Development and characterisation of chitosan nanoparticles for siRNA delivery**. *J Control Release* 2006, **115**(2):216-225.
3. Neun BW, Dobrovolskaia MA: **Method for analysis of nanoparticle hemolytic properties in vitro**. *Methods Mol Biol* 2011, **697**:215-224.
4. Russell-Jones GJ, Arthur L, Walker H: **Vitamin B12-mediated transport of nanoparticles across Caco-2 cells**. *Int J Pharm* 1999, **179**(2):247-255.
