# Supplementary material for: Preassembled complexes of hAgo2 and ssRNA delivered by nanoparticles: a novel silencing gene expression approach overcoming the absence of the canonical pathway of siRNA processing in the apicomplexan parasite Babesia microti, blood parasite of veterinary and zoonotic importance
Source: Emerg Microbes Infect. 2024 Dec 9;14(1):2438658. doi: 10.1080/22221751.2024.2438658 (PMC11721618; doi:10.1080/22221751.2024.2438658)
Supplement: Table S1.pdf [file TEMI_A_2438658_SM1974.pdf]

Table S1: ssRNA and oligonucleotide primers used in this study

Name	Primer sequence 5'--3'	Reference
B.gibsoni ssRNA* NFT	5'-Pho-rGrCrArArCrArGrCrUrArCrGrUrArUrGrUrArUTT-3'	This study
B. gibsoni ssRNA V- type ATPase	5'Pho-rCrCrUrGrArCrArUrGrArUrCrArUrUrArUrCrArArATT-3'	This study
B. gibsoni ssRNA P-type ATPase	5'Pho-rArGrUrGrArArGrArArCrUrArCrUrUrCrUrArUTT- 3'	This study
B. microti ssRNA NFT	5'Pho-rUrUrCrCrUrGrGrArArUrGrCrArUrUrGrUrArUrCTT-3'	This study
B. gibsoni NFT* RT Forward primer	5'-ACCTGCAGGCCATCTACGAC-3'	This study
B.gibsoni NFT RT Reverse primer	5'-GTGCTCGAAACCAGCGACAG-3'	This study
B. gibsoni V-type ATPase RT Forward primer	5'-CTCCTTCTCCAGAGCCATATTC-3'	This study
B. gibsoni V- type ATPase RT Reverse primer	5'-CCTCCTCGCTTATAAGTCCTTTC-3'	This study
B. gibsoni P-type ATPase RT Forward primer	5'-GGGTTGCTGCTCCATTATTTG -3'	This study
B. gibsoni P- type ATPase RT Reverse primer	5'-AGTCTCCTCCGACACTGTTA-3'	This study

*NFTRT: Nitrate format transporter Real Time PCR

*ssRNA:single stranded RNA
